# Radial dyssynchrony assessed by cardiovascular magnetic resonance in relation to left ventricular function, myocardial scarring and QRS duration in patients with heart failure

**DOI:** 10.1186/1532-429X-11-50

**Published:** 2009-11-24

**Authors:** Paul WX Foley, Kayvan Khadjooi, Joseph A Ward, Russell EA Smith, Berthold Stegemann, Michael P Frenneaux, Francisco Leyva

**Affiliations:** 1 Centre for Cardiovascular Sciences, University of Birmingham, Department of Cardiology, Good Hope Hospital, Sutton Coldfield, UK; 2 Medical School, University of Birmingham, Birmingham, UK; 3 Centre for Cardiovascular Sciences, University of Birmingham, Queen Elizabeth Hospital, Birmingham, UK; 4 Principal Scientist, Medtronic Inc, Bakken Research Center, Maastricht, NL

## Abstract

**Background:**

Intuitively, cardiac dyssynchrony is the inevitable result of myocardial injury. We hypothezised that radial dyssynchrony reflects left ventricular remodeling, myocardial scarring, QRS duration and impaired LV function and that, accordingly, it is detectable in all patients with heart failure.

**Methods:**

225 patients with heart failure, grouped according to QRS duration of <120 ms (A, n = 75), between 120-149 ms (B, n = 75) or ≥150 ms (C, n = 75), and 50 healthy controls underwent assessment of radial dyssynchrony using the cardiovascular magnetic resonance tissue synchronization index (CMR-TSI = SD of time to peak inward endocardial motion in up to 60 myocardial segments).

**Results:**

Compared to 50 healthy controls (21.8 ± 6.3 ms [mean ± SD]), CMR-TSI was higher in A (74.8 ± 34.6 ms), B (92.4 ± 39.5 ms) and C (104.6 ± 45.6 ms) (all p < 0.0001). Adopting a cut-off CMR-TSI of 34.4 ms (21.8 plus 2xSD for controls) for the definition of dyssynchrony, it was present in 91% in A, 95% in B and 99% in C. Amongst patients in NYHA class III or IV, with a LVEF<35% and a QRS>120 ms, 99% had dyssynchrony. Amongst those with a QRS<120 ms, 91% had dyssynchrony. Across the study sample, CMR-TSI was related positively to left ventricular volumes (p < 0.0001) and inversely to LVEF (CMR-TSI = 178.3 e ^(-0.033 LVEF) ^ms, p < 0.0001).

**Conclusion:**

Radial dyssynchrony is almost universal in patients with heart failure. This vies against the notion that a lack of response to CRT is related to a lack of dyssynchrony.

## Introduction

Central to the paradigm underpinning cardiac resynchronization therapy (CRT) is the concept that cardiac dyssynchrony contributes to the clinical syndrome of heart failure and that its correction translates to a clinical benefit. Accordingly, it is generally considered that pre-implant dyssynchrony is a *sine qua non *for a benefit from CRT. Conversely, it is also assumed that lack of pre-implant dyssynchrony relates to a poor outcome from CRT.

In an attempt to identify patients who were most likely to have left ventricular (LV) dyssynchrony, the CArdiac REsynchronization in Heart Failure (CARE-HF) study [1] adopted inclusion criteria of an LVEF ≤ 35% and a QRS ≥ 120 ms. It has since been recognized, however, that cardiac dyssynchrony is also present in patients with higher LVEFs [2,3] and in those with a QRS ≤ 120 ms. [4-10] In fact, cardiac dyssynchrony appears to be common in virtually all forms of heart failure, [11] where it behaves as a continuous variable, rather than as a dichotomous entity pivoting on the arbitrary cut-offs of an LVEF ≤ 35% and QRS ≥ 120 ms. [11,12]

We have previously shown that a measure of radial dyssynchrony, derived from cardiovascular magnetic resonance (CMR) tissue synchronization imaging, is a powerful predictor of mortality and morbidity after CRT in patients with a QRS ≥ 120 ms. [9] We hypothesized that intraventricular dyssynchrony is a reflection of left ventricular remodeling, myocardial scarring and impaired LV function and that, accordingly, it should be detectable in all patients with heart failure. In the present study, we have employed CMR to quantify radial dyssynchrony in a large sample of patients with ischemic (ICM) or non-ischemic (NICM) cardiomyopathy and a wide range of LVEFs and QRS durations.

## Methods

### Subjects

The study group consisted of 225 patients with heart failure who were referred for a CMR study to a single centre (Good Hope Hospital). The diagnosis of heart failure was made if symptoms of the condition were associated with objective evidence of LV dysfunction on echocardiography, or if pulmonary oedema had been documented on chest radiography in the absence of primary valvular disease. The diagnosis of ICM was made if systolic dysfunction was associated with a history of myocardial infarction [13] or if there was angiographically documented coronary heart disease (>50% stenosis in ≥ 1 coronary arteries). Late gadolinium enhancement CMR was also used to distinguish between ICM from NICM, according to Assomull et al. [14] Patients with LV dysfunction in combination with the finding of transmural or subendocardial late gadolinium uptake were classified as having ICM whereas patients with LV dysfunction and no gadolinium uptake, patchy uptake or midwall hyperenhancement were classified as having NICM. Control subjects consisted of 50 asymptomatic individuals (age 47.8 ± 15.4 yrs) who had no history of cardiac disease and a normal ECG, including a QRS duration <120 ms (78.4 ± 21.5 ms). In order to estimate the proportion of patients who might respond to CRT, a reanalysis of a previous dataset [9] was perfomed. This included 77 patients undergoing CRT. The study, which conforms to the Declaration of Helsinki, was approved by the local Ethics Committee.

### CMR

Patients were scanned using a 1.5 Tesla (General Electric Signa) scanner and a phased array cardiac coil, during repeated 8-second breathholds. A short axis stack of LV images was acquired using a steady state in free precession sequence (repetition time 3.0 to 3.8 ms; excitation time 1.0 ms; image matrix 224 × 224; field of view 36-42 cm; flip angle 45°; temporal resolution 40 ms) in sequential 8 mm slices (2 mm interslice gap) from the atrioventricular ring to apex. Left ventricular volumes, ejection fraction and mass (myocardial density = 1.05 g/cm^3^) were quantified using manual planimetry of all short-axis cine images with MASS analysis software (Medis, Leiden, The Netherlands).

### CMR-TSI

The derivation of the CMR tissue synchronization index (CMR-TSI) has been described previously. [9] Briefly, each short axis slice in the short axis stack was divided into 100 cords in a clockwise manner, from the junction between the inferior right ventricular free wall and the inter-ventricular septum. Radial wall motion was quantified for all cords at all phases in each R-R interval. This yielded up to 16,000 raw data points per patient (100 cords for each of 8 slices imaged over 20 phases. Radial wall motion data was obtained for each of 6 segments in each of, typically, 8 slices, for 20 phases (time points). Radial wall motion data (y) were fitted to an empirical sine wave function y = a + b * sin (t/RR + c). The radial motion for the 100 cords was averaged each of the 6 segments (per slice) before sine fitting. The mean segmental radial wall motion (a), the segmental radial wall motion amplitude (b) and the segmental phase shift of the maximum radial wall motion (c) were extracted from the fit. The CMR-TSI, a global measure of dyssynchrony, was expressed as the SD of all segmental phase shifts of the radial wall motion extracted from the fit. Effectively, the CMR-TSI is based on the temporal dispersion of the time-to-peak inward endocardial motion. To assess the degree to which the fitted sinus curve agreed with the observed curve, the CMR data was fitted to increasingly complex harmonic models, starting with a zero order (naïve) model and then adding first, second and third order harmonic components in a stepwise manner. Higher than third order harmonic models were not explored, so as to avoid overfitting. For each modelling step, the full model was compared to the naïve model, to decide if the more complex model improved the model significantly and if this caused a substantial change in the first order phase shift. In all cases, the first order model was statistically superior to the naïve model (p < 0.05). Second order harmonic models did increase the goodness of fit in most cases, while third order models did only improve the fit in some cases. As expected, the first order phase was not altered by adding higher order components to the fit, while the overall shape of the motion curve was better described with higher order harmonic fits.

### SLWMD

The septal-to-lateral wall motion delay (SLWMD) was defined as the time difference (in ms) between the time-to-peak inward wall motion of the septal and lateral segments, from base to apex. The observer was blinded to all other clinical details of the patients, including the outcome measures.

### Spatial Distribution of Dyssynchrony

The CMR technique described above permits visualization of radial wall motion throughout the entire LV. The phase of inward radial wall motion data derived from each short axis CMR slices were color-coded to construct bull's eye polar color maps of inward wall motion (MatLab, The Mathworks Inc, MA). The phase of inward radial wall motion were represented by colors of a spectrum ranging from blue (zero phase shift: inward motion during global systole), to green (90° phase shift: inward motion at the end of global systole) and to red (180° phase shift: inward motion during global diastole). Accordingly, the bull's eye with a homogenous blue color throughout denotes complete synchrony, whereas a bull's eye with a homogenous red color throughout denotes complete synchrony. Inhomogenous color coding denotes dyssynchrony of radial motion, with blue representing early (global systolic phase) activation and red representing late (global diastolic phase) inward radial wall motion. The spatial distribution of dyssynchrony was quantified by manually counting the number of distinct red patches (180° phase shits).

### Scar Imaging

For scar imaging, gadolinium-diethylenetriamine pentaacetic acid (0.1 mmol/kg) was given intravenously and images were acquired after 10 minutes using a segmented inversion-recovery technique in identical short-axis slices. Inversion times were adjusted to null normal myocardium (260 to 400 ms). Quantification of myocardial scarring was carried out by planimetry of enhanced tissue on late-enhancement short-axis images. Infarct volume was calculated in cm^3 ^by multiplying the planimetered area in each segment by the slice thickness. Scar volume was expressed as a % of left ventricular myocardial volume in the diastolic phase. Satisfactory images were obtained in 220/225 patients with heart failure (125/130 patients with ICM). The observer was blinded to other CMR, echocardiographic and clinical data.

### Statistical Analysis

Continuous variables are expressed as mean ± standard deviation (SD). Normality was tested using the Shapiro-Wilk test. Comparisons between continuous variables were made using the unpaired Student's *t *test, without correction for multiple comparisons. Categorical data were presented as frequencies and were compared using the Chi-squared test and Fisher's exact test. Linear and exponential regression as well as Pearson's correlation analyses were used to explore the relationship between continuous variables. For curve fitting, the fit with the greatest R^2 ^were chosen for presentation. Statistical analyses were performed using the Statview (Cary, NC) and the 2007 NCSS (Kaysville, Utah) statistical packages. A two-tailed *p *value of < 0.05 was considered statistically significant.

## Results

Compared to patients with a QRS<120 ms, patients in the QRS 120-149 ms and the QRS ≥ 150 ms groups were older, had a higher NYHA class and had higher LV volumes and a worse LVEF (Additional File 1). A greater proportion of patients in these groups had undergone a CABG. Likewise, a greater proportion of patients in these groups were on treatment with loop diuretics and spironolactone. In controls, there was no correlation between CMR-TSI and age (r = -0.16, NS).

### Dyssynchrony and QRS Duration

**Figure 1:**
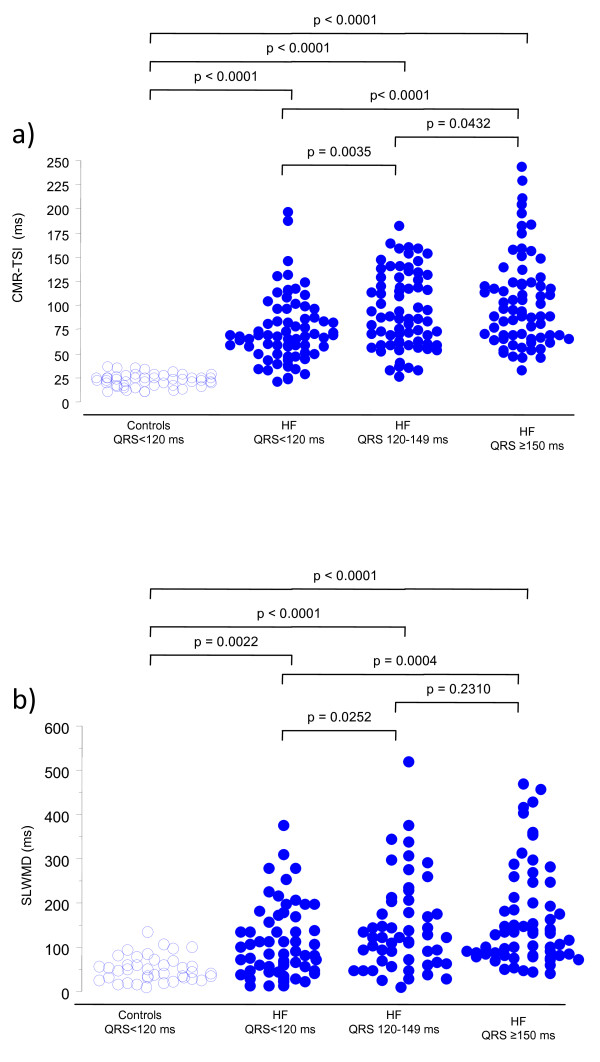
**CMR-TSI and QRS duration**. Scattergrams of a) the cardiovascular magnetic resonance tissue synchronization index (CMR-TSI) and b) the septal-to-lateral wall motion delay (SLWMD) in 50 healthy controls with a QRS <120 ms and in 225 patients with heart failure, grouped according to QRS duration.

The time-to-peak inward motion in controls ranged from a minimum of 126 to a maximum of 462 ms. The distribution of CMR-TSI in healthy controls was narrow, at 21.8 ± 6.3 ms. Compared to healthy controls, CMR-TSI was 3.4 times higher in the QRS <120 ms group (74.8 ± 34.6 ms), 4.2 times higher in the QRS 120-149 ms group (92.4 ± 39.4 ms) and 4.8 times higher in the QRS ≥ 150 ms group (104.6 ± 45.6 ms) (all p < 0.0001) (Figure 1). Adopting a cut-off of 34 ms for the definition of dyssynchrony (21.8 plus 2xSD for healthy controls is 34.4 ms), it was present in 68/75 (91%) patients with a QRS<120 ms, 71/75 (95%) patients with a QRS 120-149 ms and in 74/75 (99%) patients with a QRS ≥ 150 ms. On this basis, a total of 213/225 (95%) of patients with heart failure had dyssynchrony. If only patients in NYHA class III or IV were considered, dyssynchrony was present in 36/38 (95%) patients with a QRS<120 ms, 58/61 (95%) patients with a QRS 120-149 ms and 63/64 (97%) patients with a QRS ≥ 150 ms.

In contrast to CMR-TSI, the distribution of SLWMD in healthy controls was wider (50.8 ± 28.6 ms). Compared to healthy controls, SLWMD was 2.1 higher in the QRS <120 ms group (109.0 ± 82.6 ms p = 0.0022), 2.9 times higher in the QRS 120-149 ms group (148.0 ± 104.8 ms, p < 0.0001) and 3.3 times higher in the QRS ≥ 150 ms group (168.5 ± 113.0 ms, p = 0.0004). As shown in Figure 1b, however, there was a considerable overlap between the SLWMD in healthy controls and patients with heart failure. Adopting a cut-off of 99.4 ms for the definition of dyssynchrony (48.7 plus 2xSD [2 × 25.4 ms] for healthy controls is 99.4 ms), dyssynchrony was present in 46% in the QRS <120 ms group, 62% in the QRS 120-149 ms group and 62% in the QRS ≥ 150 ms group (168.5 ± 113.0 ms). A total of 56% patients with heart failure had dyssynchrony on the basis of a SLWMD ≥ 99.4 ms.

**Figure 2:**
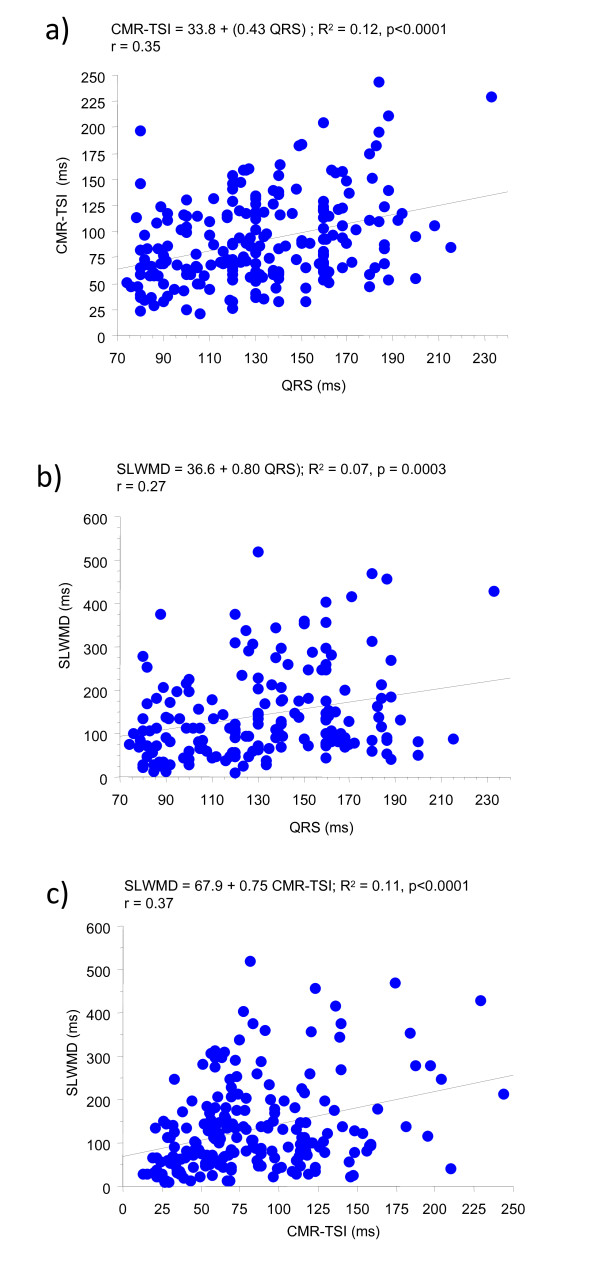
**Regression and correlation analyses of CMR-TSI, QRS duration and SLWMD**. Regression and correlation analyses of QRS duration against a) cardiovascular magnetic resonance tissue synchronization index (CMR-TSI) and b) septal-to-lateral wall motion delay (SLWMD); c) regression and correlation analysis of SLWMD against CMR-TSI. Data relates to 225 patients with heart failure.

As shown in Fig. 2, CMR-TSI correlated positively with QRS duration in patients with heart failure (r = 0.35, p < 0.0001). The correlation between SLWMD and QRS duration was also significant, although weaker (r = 0.27, p = 0.0003). As expected, there was a correlation between CMR-TSI and SLWMD (r = 0.37, p < 0.0001). The patients classified as dyssynchronous by CMR-TSI were not necessarily the same as patients classified as dyssynchronous on the basis of the SLWMD (discordance rate: 45%).

### Dyssynchrony and Left Ventricular Function

**Figure 3:**
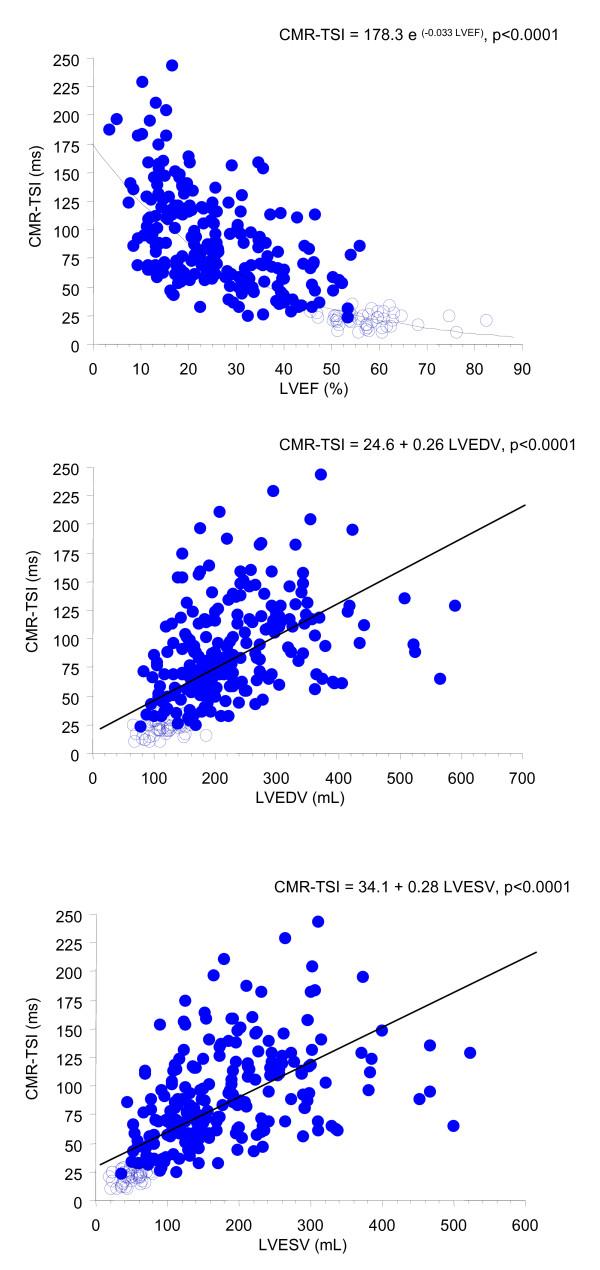
**Regression analyses of CMR-TSI against parameters of left ventricular function**. CMR-TSI = cardiovascular magnetic resonance tissue synchronization index; LVEF = left ventricular ejection fraction; LVEDV = left ventricular end-diastolic volume; LVESV = left ventricular end-systolic volume in patients with heart failure (closed circles) and in healthy control subjects (open circles).

**Figure 4:**
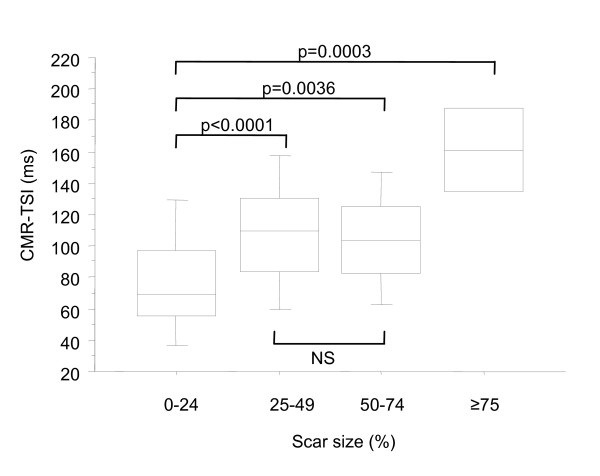
**CMR-TSI in relation to scar size**. Box-and whisker plots for C<R-TSI in relation to scar size. The five horizontal lines represent the 10^th^, 25^th^, 50^th^, 75^th ^and 90^th ^percentiles, from bottom to top.

Dyssynchrony was demonstrated in 170/174 (97%) patients with LVEF<35%. As shown in Figure 3, CMR-TSI was related to LVEF across the whole study population, according to the exponential function CMR-TSI = 178.3 e ^(-0.033 LVEF) ^(p < 0.0001). Linear relationships were observed in relation to LVEDV and LVESV (p < 0.0001). When all patients with heart failure were classified according to quartiles of scar size, the most extreme dyssynchrony was observed in patients with scars>75%. (Figure 4).

For patients such as those included in the CARE-HF study, namely patients in NYHA class III or IV with an LVEF<35% and a QRS ≥ 120 ms, dyssynchrony was present in 132/133 (99%) patients.

### Dyssynchrony and Cardiac Resynchronization Therapy

In a reanalysis of a data on 77 patients undergoing CRT,[9] (QRS duration: 150.3 ± 25.1 ms; NYHA class III [67%] and IV [33%]; LVEF: 22.6 ± 11.5%) 100% patients in the first quartile of CMR-TSI (26.5 to 80.8 ms) were responders (survival for 1 year without heart failure hospitalizations plus a reduction in ≥1 NYHA classes or a 25% increase in walking distance), compared to 75.7% in the second quartile (80.9 to 135.1 ms), 66.7% in the third quartile (135.1 to 189.4 ms) and 40% in the fourth quartile (189.5 to 243.7) (Chi-squared p = 0.0136). Deaths from cardiovascular causes at the end of the follow-up period (mean 764 days) were 0, 5/38 (13.2%), 5/15 (33.3%) and 4/5 (80%) for each quartile, respectively (Chi-squared p = 0.0002). At a cut-off of ≥ 110 ms, CMR-TSI predicted cardiovascular death with a sensitivity of 93% and a specificity of 67% (p < 0.0001). A cut-off of <81 ms was therefore taken as that which is likely to achieve a responder rate CRT of at least 75.7% following CRT. This cut-off was applied to the cohort of patients included in the present study, so as to provide an estimate of the proportion of patients who would be likely to respond to CRT. Of patients in NYHA class III or IV in the current cohort, 61/165 (37%) had a CMR-TSI <81 ms.

#### Spatial Distribution of Dyssynchrony and QRS duration

**Figure 5:**
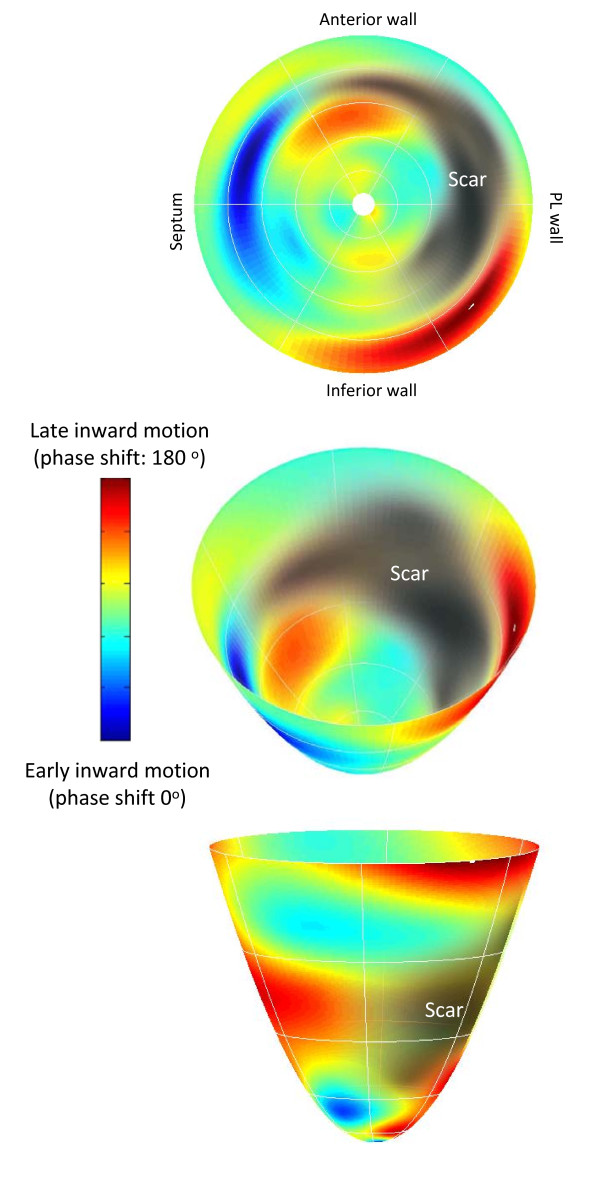
**Spatial distribution of endocardial wall motion in heart failure**. The color-encoded regional delay of radial inward motion is mapped onto a bull's eye map and three dimensional surfaces of the left ventricular model, pictured from above (middle figure) and below (bottom figure). Timing of radial inward motion is expressed as a phase delay ranging from zero to 180°. A phase delay of zero represents early ventricular motion concordant with initial electrical ventricular activation and is colour coded blue, while a phase delay of 180° denotes diastolic inward motion and is colour coded red. The posterolateral scar, shown in grey-black and imaged originally using late gadolinium-enhancement, is superimposed on the endocardial wall motion map. In the figure, the left ventricular septum is activated early in systole and the inferior wall close to the postero-lateral scar shows abnormal diastolic radial inward motion. Note the patchy distribution of wall motion throughout the left ventricle

As shown in Figure 5, patients with heart failure had a relatively heterogenous distribution of radial wall motion, with polar colour maps showing numerous 'patches' of late radial wall motion throughout the LV. In patients with heart failure, the number of patches of late radial wall motion correlated positively with QRS duration (r = 0.21, p = 0.0067), CMR-TSI (r = 0.55, p < 0.0001) and % scar volume (r = 0.16, p = 0.0434), and negatively with LVEF (r -0.52, p < 0.0001).

## Discussion

This study was undertaken in the context of an emerging 'epidemic of dyssynchrony'. [11] We have shown that cardiac dyssynchrony is present in almost all patients with heart failure. Amongst those satisfying the currently established indications for CRT, including NYHA class III or IV, an LVEF<35% and a QRS ≥ 120 ms, dyssynchrony was demonstrated in 99%. Across a spectrum of LV function and QRS duration, the relationship between radial dyssynchrony and LVEF was exponential, with extremes of dyssynchrony observed in patients with the lowest LVEF and the highest LV volumes. Moreover, the most extreme dyssynchrony was observed in patients with large myocardial scars and the longest QRS complexes. These findings indicate that cardiac dyssynchrony is a marker of LV dysfunction and myocardial injury.

### Dyssynchrony and Left Ventricular Function

We have found that an LVEF<35%, the LVEF cut-off adopted by outcome studies [1,15] and clinical guidelines of CRT, is almost invariably associated with dyssynchrony. The relationship between CMR-TSI and LVEF was governed by the exponential equation: CMR-TSI = 178.3 e ^(-0.033 LVEF)^. With respect to LV volumes, they correlated positively with dyssynchrony. Together, these findings indicate that the extremes of dyssynchrony are observed in patients with the worst myocardial function.

According to the currently accepted paradigm underpinning CRT, increasing pre-implant dyssynchrony is required for clinical benefit. Our study, however, shows that the extremes of dyssynchrony are observed in patients with the worst LVEF and the largest myocardial scars. One might therefore expect to find that increasing dyssynchrony relates to a poor outcome. Our previous demonstration that increasing dyssynchrony, assessed using CMR-TSI, relates to a high mortality and morbidity after CRT [9] is consistent with other studies of patients with heart failure showing that intraventricular dyssynchrony (assessed by tissue Doppler imaging) relates to a high risk of decompensation. [16] In this respect, Bax et al [17] found that beyond a limit of dyssynchrony (a septal-to posterior wall motion delay of 100 ms), CRT does not result in reverse LV remodeling. Together, these data invoke an alternative paradigm for CRT, namely that dyssynchrony is present in all patients with heart failure and that the lack of response to CRT is attributable to its failure to correct extremes of dyssynchrony and LV dysfunction. Our observation of a strong correlation between CMR-TSI and LV volumes suggests that the CMR-TSI reflects the severity of left ventricular remodeling. Accordingly, patients with a high CMR-TSI may be less likely to respond to CRT simply because left ventricular dilatation is so excessive that it is not susceptible to the reverse remodeling effects of CRT.

### Dyssynchrony and QRS duration

Using tissue Doppler imaging and a 12-segment model, Yu et al [5] demonstrated systolic dyssynchrony in only 73% of patients with heart failure and a QRS > 120 ms. Similar findings have emerged from another tissue Doppler study using a six basal and six middle LV segment model. [18] In the present study, however, 99% of patients with heart failure, in NYHA class III or IV, an LVEF<35% and a QRS>120 ms had dyssynchrony. With respect to patients with heart failure and a QRS<120 ms, tissue Doppler imaging studies have shown that dyssynchrony is present in 36% to 65% of patients, [5,6,19] depending on the particular method employed. In contrast, we have found dyssynchrony in 91% patients with a QRS<120 ms. Several factors may contribute to our finding of a greater prevalence of dyssynchrony in patients with heart failure. Firstly, CMR provides imaging of the entire LV, whereas echocardiography has a limited window. Secondly, CMR-TSI is based on radial wall displacement, rather than longitudinal velocities. Thirdly, tissue Doppler imaging has high interobserver variability, (interobserver coefficient of variation of 33.7% for 12-segment model) [20] whereas this is low (9%) for CMR-TSI. [9] Importantly, large discrepancies between different echocardiographic methods used to detect dyssynchrony are well recognized. [20,21]

Prolonged QRS duration results in late myocardial activation, usually in the posterolateral LV segments. A correlation should therefore be expected between QRS duration and measures of LV dyssynchrony. Although a correlation between QRS duration and cardiac dyssynchrony has been shown using radionuclide phase analysis, [8] studies using tissue Doppler have found only a weak correlation [6] or no correlation at all [5,18]. Using two-dimensional speckle tracking, Donal et al have recently shown that QRS duration correlates with radial dyssynchrony in patients with heart failure due to NICM. [22] We have found that, in patients with heart failure, QRS duration does indeed correlate with both SLWMD and with CMR-TSI. Together, these studies suggest that the finding of a correlation between QRS duration and dyssynchrony measures depends upon the imaging method employed.

Using colour-coded bull's eye maps of radial wall motion, we have shown that, in patients with heart failure, dyssynchrony is distributed unevenly throughout the LV. Moreover, the spatial heterogeneity of dyssynchrony, quantified in terms of the number of patches of late radial wall motion in bull's eye maps, correlates positively with QRS duration and scar volume, and negatively with LVEF. These findings are relevant to studies showing that CRT is more effective when LV leads are deployed in areas of delayed contraction. [23,24] Importantly, however, our findings of a patchy distribution of dyssynchrony might also account for the apparent superiority of CRT using multi-site over single-site LV pacing. [25] It might also account for the inordinately high interstudy variabilitites observed with echocardiographic measures of dyssynchrony, [20] which are usually derived from two-dimensional imaging.

### CMR-TSI and SLWMD

We have found significant differences in the SLWMD between healthy controls and patients with heart failure. Whilst only 56% of patients were classified as having dyssynchrony on the basis of the SLWMD (≥ 99.4 ms), 95% had dyssynchrony on the basis of the CMR-TSI (>34 ms). This suggests that CMR-TSI, which is based on wall motion data from the entire LV, is more sensitive at detecting dyssynchrony than SLWMD, which is based on wall motion data from the septal and lateral segments only. Importantly, the overlap between the values for healthy controls and patients with heart failure was less for CMR-TSI than for SLWMD. It would appear, therefore, that the ability to detect dyssychrony and to differentiate between healthy controls and patients with heart failure depends on the sensitivity of the method used to measure it.

### Clinical Implications

Dyssynchrony was demonstrated in virtually all patients with heart failure who satisfied the currently established indications for CRT. Our findings extend the current paradigm of CRT to patients with heart failure and a QRS ≤ 120 ms. Although the recently reported RethinQ study showed no symptomatic benefit from CRT in implantable cardioverter defibrillator recipients with a QRS <120 ms, [26] Yu et al observed that CRT alone led to improvements in NYHA class, 6-min walking distance, LVEF and mitral regurgitation. Crucially, these effects were reversed by withholding CRT after 4 weeks. [27] Similar benefits from CRT in patients with heart failure and a narrow QRS <120 ms have been shown by other studies. [28] Our finding that the majority (91%) of patients with a QRS<120 ms have radial dyssychrony provides a rationale for applying CRT to this patient population.

In an attempt to identify the cut-off of dyssynchrony below which one should expect a reasonable response to CRT, we reanalysed data from a previous study of patients undergoing CRT. [9] In this reanalysis, we identified a CMR-TSI < 81 ms as the cut-off below which one should expect a responder rate of at least 75.7%. These findings, however, raise the possibility that on the basis of the CMR-TSI, more than a third of patients with heart failure are likely to derive a reasonable response from CRT. Admittedly, this requires confirmation in a study of CRT that includes patients with a wide range of QRS durations.

Further randomized studies are needed to determine whether the less severe dyssynchrony observed in these patients is an appropriate substrate for CRT. We should consider, however, that factors other than dyssynchrony, such as myocardial viability, also govern the response to CRT. Comparison of CMR-derived measures of dyssynchrony with respect to response after CRT is required. Prominent amongst other measures is the circumferential uniformity ratio estimate (CURE), which has recently been shown to predict symptomatic response to CRT. [29] The CMR-TSI offers a possible advantage over the CURE index, as it is based on the entire LV, whereas the CURE index is based on an averaged slice estimate.

## Conclusion

We conclude that cardiac dyssynchrony is virtually synonymous with heart failure. The finding that dyssynchrony is present almost all patients with heart failure who satisfy the currently established indications for CRT casts doubt notion that a lack of response to CRT is related to a lack of dyssynchrony. Further studies are needed to determine what level of dyssynchrony translates to a clinical benefit from CRT, including those with a QRS <120 ms. The additional finding that dyssynchrony is distributed in a patchy fashion throughout the left ventricle provides a rational basis for multisite LV pacing.

## Competing interests

The authors declare that they have no competing interests.

## Authors' contributions

PWXF collected and analyzed data and drafted the manuscript. KK collected data. JW collected data. REAS recruited and carried out implantations and contributed intellectually to the manuscript. BS carried out image and data analyses and also contributed to statistical analysis and drafting of the manuscript. MP conceived part of the study and helped draft the final manuscript. FL conceived the hypothesis, designed the study, collected and analyzed data and contributed to drafts and final version of the manuscript. All authors read and approved the final manuscript.

## Supplementary Material

Additional file 1 **Table 1**. Clinical, Electrocardiographic and CMR Characteristics for Patients Included in the Study, Grouped According to QRS Duration Click here for file
