# Supplementary material for: Radial dyssynchrony assessed by cardiovascular magnetic resonance in relation to left ventricular function, myocardial scarring and QRS duration in patients with heart failure
Source: J Cardiovasc Magn Reson. 2009 Nov 24;11(1):50. doi: 10.1186/1532-429X-11-50 (PMC2789061; doi:10.1186/1532-429X-11-50)
Supplement: Additional file 1 — Table 1. Clinical, Electrocardiographic and CMR Characteristics for Patients Included in the Study, Grouped According to QRS Duration [file 1532-429X-11-50-S1.DOC]

**Table 1**. Clinical, Electrocardiographic and CMR Characteristics for Patients Included in the Study, Grouped According to QRS Duration.

**QRS duration p**

**A B C B vs A C vs A C vs B**

**<120 ms 120-149 ms ≥150 ms**

N 75 75 75

Age, yrs 59.8 ± 11.6 66.5 ± 11.8 65.0 ± 11.9 0.0006 0.0078 NS

Gender, male (%) 57 (76) 55 (73) 56 (75) NS NS NS

NYHA class, n (%)

I 27 (36) 5 (7) 7 (9)

II 10 (13) 9 (12) 4 (5)

III 28 (37) 46 (61) 48 (64)

IV 10 (13) 15 (20) 16 (21)

As continuous variable * 2.28 ± 1.09 2.95 ± 0.77 2.97 ± 0.81 <0.0001 <0.0001 NS

**Etiology, n (%)**

ICM 41 (55) 43 (57) 46 (61) NS NS NS

NICM 34 (45) 32 (43) 29 (39) NS NS NS

**Co-morbidity, n (%)**

Diabetes mellitus 7 (9) 15 (20) 8 (11) 0.0648 NS NS

Hypertension 21 (28) 20 (27) 17 (23) NS NS NS

CABG 5 (7) 18 (24) 13 (17) 0.0032 0.0444 NS

**Medication, n (%)**

Loop diuretics 43 (57) 59 (79) 62 (83) 0.0051 0.0007 NS

ACE-I or ARB 64 (85) 66 (88) 62 (83) NS NS NS

Beta-blockers 38 (51) 38 (51) 50 (67) NS NS NS

Spironolactone 11 (15) 31 (41) 31 (41) <0.0001 <0.0001 NS

**ECG variables**

Sinus rhythm, n (%) 64 (85) 61 (81) 56 (75) NS NS NS

Atrial fibrillation, n (%) 11 (15) 14 (19) 19 (25) NS NS NS

QRS duration, ms 94.2 ± 12.4 129.1 ± 10.6 169.6 ± 17.7 <0.0001 <0.0001 <0.0001

**CMR variables**

LVEDV, cm^3^ 184.5 ± 64.9 230.1 ± 88.9 266.6 ± 102.8 0.0016 <0.0001 0.0112

LVESV, cm^3^ 134.9 ± 63.0 181.2 ± 87.8 218.1 ± 100.4 0.0011 <0.0001 0.0087

LVEF, % 30.7 ± 13.2 24.6 ± 11.8 21.3 ± 9.2 0.0013 <0.0001 NS

% scar volume 21.5 ± 23.6 17.4 ± 21.3 18.3 ± 20.1 NS NS NS

p values refer to differences between the QRS<120 ms and the QRS≥120 ms groups. Continuous variables are expressed as mean ± SD. *, For functional class, statistical analyses were applied to NYHA class, expressed as a continuous variable. NYHA=New York Heart Association class. ICM = ischemic cardiomyopathy; NICM = non-ischemic cardiomyopathy; CABG = coronary artery bypass grafting; ACE-I=angiotensin converting enzyme inhibitors; ARB=angiotensin II receptor blockers; LVEF = left ventricular ejection fraction; LVEDV = left ventricular end-diastolic volume; LVESV = left ventricular end-systolic volume.
